# Drug Disposal and Ecopharmacovigilance Practices in the Krowor Municipality, Ghana

**DOI:** 10.1155/2022/7674701

**Published:** 2022-12-30

**Authors:** Yvonne Yirenkyiwaa Esseku, Priscilla Kolibea Mante, Alex Nii Oto Dodoo, Eric Woode

**Affiliations:** ^1^Department of Pharmacology, Kwame Nkrumah University of Science and Technology, Kumasi, Ghana; ^2^Ghana Standards Authority, Accra, Ghana; ^3^Department of Pharmacology and Toxicology, University of Health and Allied Sciences, Hohoe, Ghana

## Abstract

**Introduction:**

The use of medicines is a ubiquitous practice for the management of healthcare conditions. In the delivery of healthcare, medicines may remain unused and may expire within the various stakeholders in the pharmaceutical value chain. If these unused and expired medicines are not disposed of properly, they may result in the concentration of pharmaceuticals in environmental media contaminating food sources for humans and animals. Implementation of ecopharmacovigilance strategies will reduce the quantities of pharmaceuticals in the environmental media, reduce the potential for inadvertent consumption by humans and animals, and reduce potential pharmacological effects on the environment, humans, and animals. The drug disposal flow diagram (DDFD) provides an effective way of assessing the most cost-effective strategies to reduce environmental contamination.

**Method:**

A combined method of desk study and questionnaires, both structured and unstructured was used. The desk study reviewed the institutional arrangements for the regulation of disposal of pharmaceutical waste in Krowor. The questionnaires were used to gather information from community members, community pharmacies, and pharmaceutical manufacturers in Krowor.

**Results:**

The drug disposal flow diagram shows that up to 96% of pharmaceuticals are handled and disposed of in ways that are harmful to the environment with only 4% being handled in ways that are environmentally friendly. Forty-nine percent (49%) of generated pharmaceutical waste ends up in the local and surrounding areas, 21% contaminates the drainage system and 25% is discharged into receiving waters. *Discussion*. The DDFD for Krowor shows that engagement with community members and institutional healthcare service providers and strategies that result in separation of pharmaceutical waste from general household waste will reduce the quantities of pharmaceuticals that end up in the environmental media.

**Conclusion:**

The DDFD will support the effective implementation of ecopharmacovigilance (EPV) strategies.

## 1. Introduction

The World Health Organization (WHO) estimates that more than 50% of all the medicines are prescribed, dispensed, and sold inappropriately and half of all patients fail to take the medicines correctly [1]. The result of such inappropriate prescribing, dispensing, and use of medicines is that patients are likely to have various unused medicines in their possession which will require disposal [2]. It must be noted that even where medicines are prescribed and dispensed appropriately, it is possible for consumers to have medicines that ought to be disposed of.

This may be because medicines are changed by the prescribers before the patients complete the regimen [2]. It may also be because dispensing practices mean that there are some amounts of medicines that are given as excess to consumers to make up for spillages of some liquid dosage forms or losses from solid dosage forms that are dropped before administration [2]. Prepackaged liquid dosage forms, by their presentation are also likely to have some excess doses which will not be consumed even by the most conscientious patient [2].

When medicines are not disposed of properly, they end up concentrated in various environmental media. This is the same with residue from empty medicine containers which are not disposed of properly [3]; the presence and concentration of pharmaceuticals in the environment results in various effects on organisms that depend directly on the contaminated environment for survival. Kummerer and Velo have undertaken a comparison of the effects of medicines in humans and the environment [4, 5]. Both Kummerer and Velo show that in humans, there is generally a specific reason for which medicines are taken.

The number of medications being taken will normally be known and limited to only a few at a time. This limits the exposure and the effects, whether or not the effects are intended to only the person(s) taking the medication. On the other hand, when medicines enter the environment, the exposure is less specific and therefore more diffuse. The effects of such exposure will also be diffuse. Again, the biotransformation of most administered pharmaceutical agents may generally be well understood as it applies to one organism. In the environment, however, the biotransformation takes place in various types of organisms [4]. The biochemical processes of biotransformation in these organisms may not be clearly understood and the affected organisms may not be known.

The effects of the presence of pharmaceuticals in the environmental media are, thus, not clearly understood and may be complex and long-term on environmental flora and fauna. Ecopharmacovigilance (EPV) has been said to describe the science and activities associated with the detection, evaluation, understanding, and prevention of adverse effects of pharmaceuticals in the environment [6]. The potential for inadvertent effects of pharmaceuticals to occur in the environment brings up a need for EPV wherever there is a potential for environmental media to be exposed to these products.

Authors have previously designed the drug disposal flow diagram (DDFD); a tool for assessing the presence of pharmaceuticals in the environmental media [2]. This tool is particularly appropriate to ascertain the interventions that will be appropriate in specific settings. The importance of the tool lies in the fact that although several strategies have been implemented in different settings with the aim of dealing with pharmaceuticals in the environmental media, but not all strategies may not be appropriate for all settings. In resource constrained environments, where it may not be possible to deploy several strategies, the tool provides a focus of investment to ensure that resources are properly utilised and value for money is obtained in deploying interventions.

**Figure 1:**
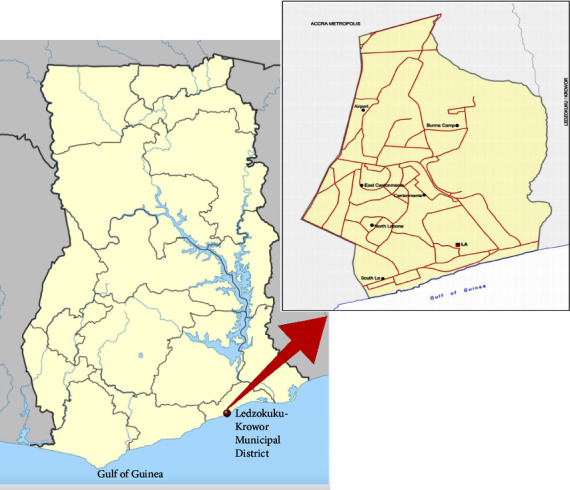
Map of Ghana and communities in the Krowor municipality (by nordnordwest and manny 9455 licensed under CC BY-SA 3.0 and 4.0).

Therefore, the aim of the study was to utilise information from various stakeholders involved in the handling and use of medicines in the Krowor Municipal Assembly area (Figure 1) to design a drug disposal flow diagram to ascertain appropriate drug disposal practices for the municipality.

## 2. Methods

The study combined a desk analysis and surveys. In the desk analysis, studies and literature on pharmaceuticals in the environment were reviewed. Structured surveys were used to assess how unused and unwanted pharmaceuticals are handled by consumers and community pharmacies. A semistructured survey was used to gather information from regulators and pharmaceutical manufacturers on the disposal practices. Samples of the questionnaires used in data collection are attached as appendices. The study was approved by the Humanities and Social Science Research Ethics Committee, Kwame Nkrumah University of Science and Technology (HuSSREC no. 429/2019).

### 2.1. Desk Study

The desk study involved review and analyses of the relevant literature. Eight national policies and guidelines relevant to health, environmental sanitation, and management of health care waste were reviewed. Six Acts of Parliament were also reviewed. These enactments govern the activities of the Food and Drugs Authority, the Environmental Protection Agency, and District Assemblies when it comes to the handling of medicines and medical waste.

A search was conducted on online learning platforms including Google Scholar and Research Gate using key phrases such as “medicines in environment,” “environmental impact of medicines,” “development of resistance to medications,” and “disposal of medicines.” After an initial review of responses, fifteen articles were determined as relevant to the research. These articles were reviewed further.

### 2.2. Discussions with Krowor Municipal Assembly

Discussions were held with the Municipal Environmental Health Officer (MEHO) in September, 2019. The discussions were aimed at gathering information with respect to the following as they pertain to the Krowor municipality: health facilities and their categorization, disposal of waste from health facilities and disposal of waste from households.

### 2.3. Structured Survey of Community Members

#### 2.3.1. Survey Site

The site for the survey was community pharmacies in the Krowor Municipal Assembly (KroMA). Participants were recruited as they left community pharmacies after obtaining pharmaceutical products and services. The pharmacies from which community members were surveyed are scattered across the assembly and are in residential, commercial, and industrial sections in the assembly.

The municipality is one of the thirty-seven (37) new municipalities created with Legislative Instrument (LI) 2318. It was carved from the erstwhile Ledzokuku-Krowor Municipal Assembly in the Greater Accra Region and has its capital as Nungua.

#### 2.3.2. Population and Sample Size

The municipality is in the Greater Accra Region. It is located on the coast between the Tema West Municipality and Ledzokuku Municipality. The assembly covers a land area of 27.58 km^2^ [7]. The main economic activity in the municipality is commerce comprising 42.5%. This is followed by manufacturing (15.8%) and agriculture (10.1%). Approximately, sixty percent (60%) of residents in the municipality with indigenes and forty percent (40%) being settlers Ibid.

The population of the Krowor Municipal Assembly area is estimated at 137,620. This estimate was arrived at using projections of district populations from the Ghana Statistical Service [8].

The calculation was done as set out in the supplementary material (S1). The sample size for data collection was determined using the following formula:

(1) $$\begin{array}{c} Sample\;Size=\left(〖\left(z-score\right)〗{}^{\wedge }2\times StdDev\times \backslash \left(1-StdDev\right)\right)/〖\left(margin\;of\;error\right)〗{}^{\wedge }2. \end{array}$$

The confidence level (*z*-score) of 95%, a margin of error of 5%, and a standard deviation of 0.5 were used for this survey. The calculation gave a figure of 384 as the ideal sample size. A sample size of four hundred (400) was used for the study. This is to allow for the results to be sufficiently strong to be extrapolated to the whole population. To avoid bias, the participants were randomly contacted as they left community pharmacies from which they had accessed health service.

#### 2.3.3. Data Collection Techniques

A structured questionnaire was used in the collection of data. The data captured were, thus, quantitative in nature and did not allow the capture of perceptions of participants. The tool used in data collection is set out in the supplementary material. Participants were contacted as they left the community pharmacies after receiving pharmaceutical service, and the purpose of the study was explained to them. Verbal informed consent was sought from the participants. Following receipt of consent, the questionnaire was administered by the data collector. In instances where the respondent expressed a preference, the questionnaires were self-administered by the respondent with guidance from the data collector when necessary.

### 2.4. Structured Survey of Pharmacies

#### 2.4.1. Survey Site

The sites for this survey were community pharmacies in KroMA. These included retail pharmacies and facilities that are registered as wholesale/retail.

#### 2.4.2. Population and Sample Size

There are thirty-three (33) community pharmacies in KroMA which provide pharmaceutical services on a retail basis. An additional two (2) pharmacies provide wholesale and retail services from their premises. The relevant population for administering the questionnaire was, thus, thirty-five. A response rate of fifty percent (50%) was estimated as sufficient to make inferential analysis.

#### 2.4.3. Data Collection Techniques

A structured questionnaire was used in data collection. The tool used is set out in the supplementary materials (S2–S4). The questionnaire was submitted to all the facilities. Follow-up calls were made to take back completed forms.

#### 2.4.4. Discussions with Pharmaceutical Manufacturers in Krowor

Letters were sent to the Chief Executive Officers of the pharmaceutical manufacturers requesting an interview to gather the relevant information. A discussion guide (See supplementary material) was used to guide discussion and facilitate data collection.

### 2.5. Data Analysis

Data from the surveys were entered into Microsoft Excel for analysis. Results for categorical and continuous variables were, respectively, presented as numbers (percentages) and the mean ± standard deviation (SD).

## 3. Results

### 3.1. Results from Discussion with Krowor Municipal Assembly

#### 3.1.1. Health Facilities and Their Categorizations

The Municipal Environmental Health Officer stated the following as some of the categories of the health facilities in Krowor from which pharmaceutical waste may be discharged: One polyclinic, ten clinics, two Community Health Planning Services (CHPS) compounds, and an indeterminate number of herbal clinics and shops [9]. In addition to these, there are twelve wholesale pharmacies, thirty-three retail pharmacies, and two facilities that operate and wholesale/retail pharmaceutical outlets in the Krowor municipality according to the data obtained from the Pharmacy Council of Ghana. All these are sources from which pharmaceutical waste may be discharged into environmental media.

#### 3.1.2. Disposal of Waste from Health Facilities

The local assembly is involved in the disposal of waste from hospitals, clinics, and CHPS compounds in the municipality [9]. When these health care facilities have medicines and other pharmaceutical waste to dispose of, an application is made to the assembly. The assembly conducts an inspection, and an assessment is made with respect to the methods of disposal to be used. The methods which are normally used are burning, burying in the ground, dissolving in water, and discharging into municipal drains. These methods are used for waste that is determined to be nonhazardous [9]. Where the waste is determined to be hazardous, specialized waste collection companies are contacted to handle the waste [9]. Determination of hazardous or nonhazardous waste is based on definitions set out by the Health Care Waste Management Policy of the Ministry of Health, Ghana. The policy defines hazardous waste as including infectious (including pathological and sharps), radioactive, and pharmaceutical waste [9].

#### 3.1.3. Waste from Households in the Municipality

In the disposal of medicines by households and individuals in general, guidance from EPA indicates that households and individuals are required to separate any medicines from the general waste. The unused and expired medicines are to be given to the waste collection company when they pick up the general waste [10].

According to the District Assembly (DA) respondent, in Krowor, household waste in the municipality is collected by registered waste collectors. The registered waste collectors are persons who have been assessed as able to transport the collected waste. Community members register with the waste collectors to ensure regular collection. Fees for collection are paid by the community members. Following collection, the household waste is disposed of in designated dump sites in the region. The Krowor Municipality has no dumping site and so utilises dumping sites in other assemblies within the region. It is the responsibility of the Assembly to ensure that there are dumping sites for the disposal of household waste in Krowor. The DA respondent, however, indicated that household waste is not segregated in any way [9]. Thus, members of households will dispose of their expired and unused medicines with the general waste.

### 3.2. Community Members in Krowor

#### 3.2.1. Demographics of Respondents

**Figure 2:**
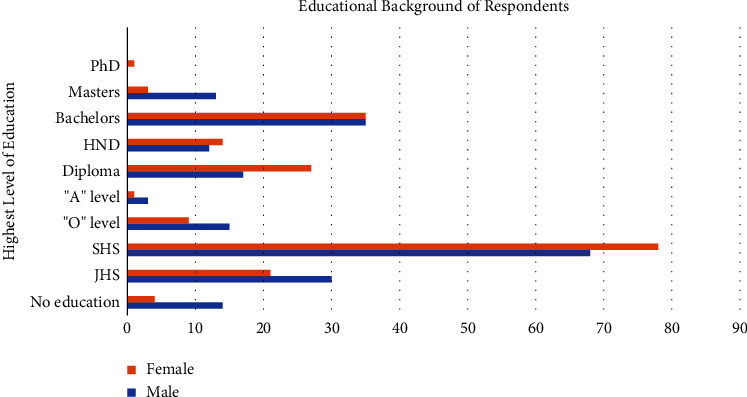
Educational background of the survey respondents in the Krowor municipality comparing gender differences. Data represent the means for each group (*n* = 400). (PhD: doctor of philosophy; HND: higher national diploma; SHS: senior high school; and JHS: junior high school).

In the conduct of the survey, community members were contacted as they exited community pharmacies from which they had obtained pharmaceutical service. Four hundred community members participated in the survey. Of these, two hundred and seven (52%) were male and one hundred and three (48%) were female. The educational background of respondents cut across the spectrum from no education background to one PhD.  Figure 2 shows the distribution of the educational background of survey participants disaggregated by gender. Respondents ranged in age from sixteen (16) years to seventy-two (72) years of age. The modal and median age range of respondents was twenty-six (26) to thirty (30) years of age which accounted for twenty-three percent (23%) of all respondents participating in the survey. The age bracket of respondents correlates with the age bracket of active persons in the community.

#### 3.2.2. Handling of Medicines by Community Members

The survey sought to assess behavior of community members concerning the handling of medicines. In this regard, participants were asked about medications they have from previous visits to the pharmacy, medications they have kept using in the event of emergencies, and medications they must use for conditions they may still be treating.

#### 3.2.3. Medicines from Previous Visits

Consumers may not be able to consume all the medications dispensed to them during a particular visit. Such unused medicines may in the future be used for some other purpose. They sought to ascertain the percentages of communities who have medicines left over from previous visits to health care facilities. When asked whether they were able to complete all medications supplied when they seek health care, about eighty-one percent (81%) of males and seventy-nine percent (79%) of females responded in the affirmative. When asked whether they had medications at home from a previous visit thirty-seven percent (37%) of males and thirty-four percent (34%) of females indicated that they did, with the other 63% of males and 66% of females indicating that they did not have any medications from a previous visit. This does not align with the answers given by respondents with respect to their ability to complete all medications.

#### 3.2.4. Medicines for Emergency and Other Use

**Figure 3:**
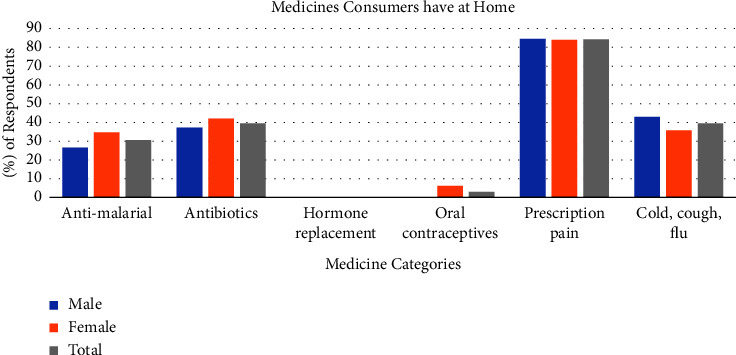
Major types of medications consumers in the Krowor municipality admit to having at home for emergency use. Data represent the means for each group (*n* = 400).

Participants were questioned on medications they have at home for use in emergencies. To this, 81% of males and 83% of females answered in the affirmative. When questioned further about the types of medications they have at home, up to 84% indicated that they had medications which had been prescribed for pain at home. Participants also identified other categories of medicines they have at home as set out in Figure 3. Significant numbers of consumers reported having antimalarial agents, antibiotics, medicines prescribed for pain. and medicines for cold, cough, and flu. The category of medicines for cold, cough, and flu were of interest because most preparations for the management of these ailments contain medications for pain relief. One category of interest was medications containing hormones such as those for hormone replacement or as oral contraceptives. Although none of the respondents indicated that they had any medications for hormone replacement, 3% of respondents (all female) indicated that they had oral contraceptives.

#### 3.2.5. Disposal of Unused and Expired Medicines

**Figure 4:**
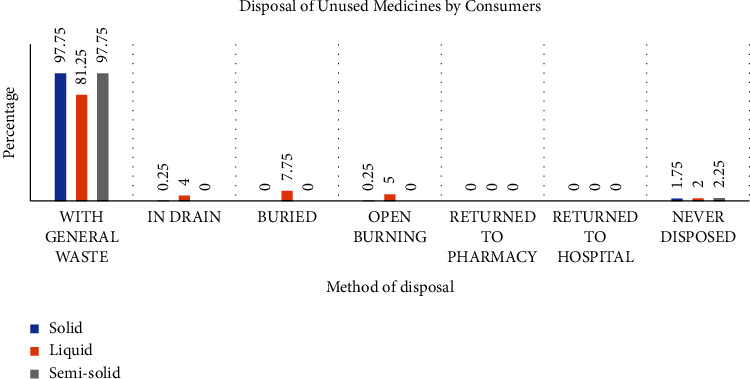
Methods of disposal used by community members in Krowor municipality for different drug dosage forms. Data represent the means for each group (*n* = 400).

The study sought to ascertain the actions community members take when they need to dispose of medicines. To this end, respondents of the survey were asked about the means by which they dispose of medicines when the need arises. About ninety-eight percent (98%) of respondents indicated that they add solid and semisolid medicines to the general waste for disposal. With respect to liquid dosage forms, about eighty-one percent (81%) of respondents said they add them to the general waste, four percent (4%) pour them down the drain, about eight percent (8%) bury them, and five percent (5%) use open burning when they must dispose of these medicines. None of the respondents return their unused and expired medicines to the pharmacy or hospital for disposal.  Figure 4 shows the details of the disposal methods used by community members for different dosage forms.

### 3.3. Pharmacies in Krowor

#### 3.3.1. Demographics of Respondents

The responding facilities were in residential and commercial sections in the assembly. The residential and commercial sections provided fifty percent (50%) each of responding facilities in Krowor. Although there is a light industrial area in KroMA, there was no response from facilities in that area. Thirty-five percent (35%) of pharmacists who responded were female and sixty-five percent (65%) were male. The ages of respondents ranged from twenty-five (25) to fifty-eight (58) years with three respondents refusing to provide details of their age. The years of experience also ranged over a spectrum of one year to thirty-nine (39) years of practice as pharmacists.

#### 3.3.2. Unused and Expired Medicines

Community pharmacies may have some expired and unused medicines from time to time. These must be dealt with using different methods of disposal. To gather information on how these medicines are handled, respondents were asked various questions relating to storage, categories of unused and expired medicines, and means of disposal. Participants were asked whether there is any form of categorization of unused and expired medicines for storage. Participants were asked whether the facilities had ever had to deal with unused and expired medicines. Only one facility had not unused and expired medicines.

**Figure 5:**
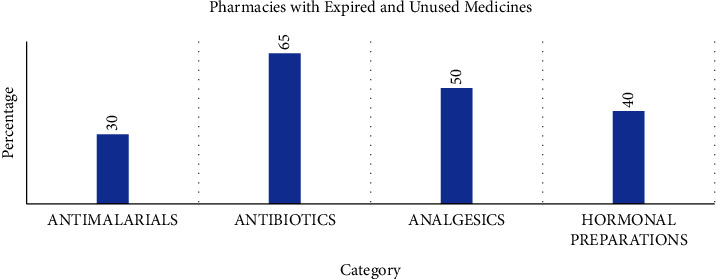
The percentage of pharmacies holding medicines requiring disposal in the Krowor municipality. Data represent the means for each group (*n* = 35).

With respect to handling of unused and expired medicines, participants were asked about whether medicines are sorted before disposal. Of the facilities that had dealt with unused and expired medicines, fifty-three percent (53%) stated that unused and expired medicines were sorted out for storage before disposal. The respondents were asked about the presence of unused and expired medicines of specific categories. These categories were antimalarial preparation, antibiotics, analgesics, and hormonal preparations. The percentages of pharmacies who had these medicines are shown in Figure 5.

#### 3.3.3. Disposal of Medicines by the Pharmacies

The study sought to identify the specific ways in which the facilities disposed of unused and expired medicines, wherever applicable. Respondents were therefore asked whether they had ever disposed of any medications in the past. The methods employed for the disposal of these medicines were also noted. The results showed that eighty percent (80%) of facilities had disposed of medicines in the past.

Of the facilities that had disposed of medicines in the past, up to 75% indicated that solid dosage forms were sent to the Food and Drugs Authority, Ghana (FDA) for disposal. The percentage of facilities that sent liquid dosage forms and semisolid dosage forms were about 62% and 69%, respectively. None of the respondent facilities had buried any type of unused and expired medicines and none had used drains for disposing of solid and semisolid dosage forms of medicines. The facilities also did not use open burning when disposing of liquid dosage forms.  Table 1 sets out the breakdown.

Although most respondent pharmacies indicated that they handed over their medicines to the FDA when there is the need for disposal, forty percent (40%) of respondents did not know the methods used by the FDA during disposal of medicines.

**Figure 6:**
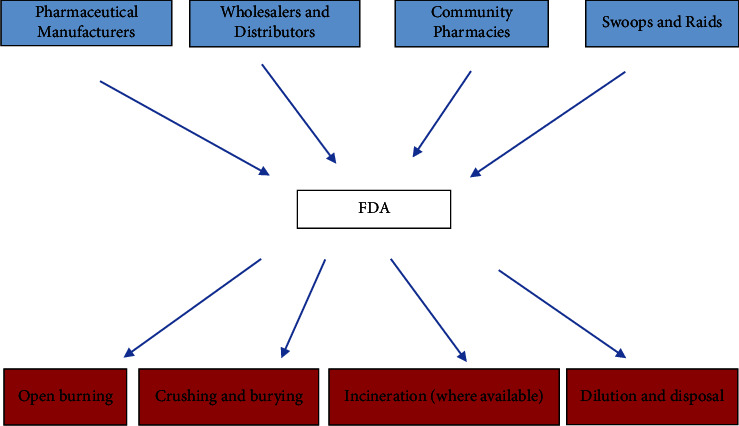
Flow of medicines during regulated disposal–from source to sink. Source: author (from KII with FDA).

Figure 6 provides an illustration of the flow of medicines during regulated disposal supervised by the FDA. Regulated disposal of medicines by the FDA involves disposal of medicines collected by the Authority through various means. The FDA regularly receives medicines to be disposed off from pharmaceutical manufacturers, wholesalers, and distributors as well as community pharmacies. Regular “swoops and raids” organized by the FDA to clamped down on illegal distribution of medicines by unauthorized individuals and outfits, is also a common source of medicines that undergo regulated disposal.

#### 3.3.4. Pharmaceutical Manufacturers in Krowor

There are three (3) pharmaceutical manufacturing concerns in KroMA. All were contacted, but one declined to participate in the study. The institutions that participated in the study produce the finished products from raw materials. They do not undertake repackaging of already formulated products. The ranges of formulations by these manufacturers are liquid oral dosage forms, solid oral dosage forms, ointments, and cream. The discussions showed that the manufacturers have totally different systems of disposal. For the purposes of this report, the institutions will be referred to as A and B to maintain confidentiality of the responses.

#### 3.3.5. Disposal of Active Pharmaceutical Ingredients (APIs) and Medicines

Institution A has no organizational protocols in place for disposing of APIs or medicines. This is because all their APIs are usually used up and the organization estimates that only about “0.01% of the raw materials” end up as waste which needs to be disposed of. Such waste is handled as they do other solid waste in the organization. On some rare occasions where there is the need to dispose of APIs or medicines, the disposal are supervised by the FDA and Environmental Protection Agency (EPA).

Institution B, on the other hand, has standard operating procedures (SOPs) for when the need arises for the disposal of APIs. In such instances, the FDA is notified to inspect, quantify, and supervise the destruction of the APIs. Such destruction is undertaken off the premises of the manufacturing facility. The organization is thus not involved in that process. Where their SOPs so require, the EPA is contacted when APIs or finished products must be disposed of.

#### 3.3.6. Treatment of the Effluent

The effluent generated in Institution A goes to a storage reservoir outside the factory premises. The effluent is pumped through digesters and treated. The treated water is rechanneled for use in the factory washrooms. This treatment of effluent does not depend on the category of medicine in question. All effluents, whether from beta lactam or non-beta lactam production, are treated in the same way and treated together in the same system. Institution B has a wastewater treatment plant (WWTP) on-site which is used in the treatment of all effluent. Production in institution B does not include antibiotics. There is no indication of reuse of the water after treatment in Institution B.

#### 3.3.7. Drug Disposal Flow in Krowor Municipality

The DDFD designed by the author [2] is a useful tool in assessing the flow of pharmaceuticals in the Krowor municipality. In applying the tool to Krowor, the participants in the pharmaceutical value chain were identified as manufacturers, institutional care service providers, community pharmacies, and consumers. The information obtained from these participants during the study is utilised in the matrix designed by Esseku [2]. From this matrix, the DDFD for Krowor is obtained. This provides a pictorial presentation of the way in which pharmaceuticals flow into environmental media in Krowor. This provides information on the most cost-effective means of dealing with pharmaceuticals in the environment in the municipality. The process of flow of pharmaceuticals can be described (See Supplementary Material: SM2). This process captures the direction of flow of pharmaceuticals in Krowor.

In designing the DDFD, the data collected are used to estimate the quantities of pharmaceuticals generated by the different categories of participants in the pharmaceutical value chain [2]. With regards to the manufacturers and institutional care service providers, percentages were determined based on information obtained from the municipal assembly; the municipal assembly collected and disposed of the waste from these facilities. With regards to the community pharmacies and consumers, percentages were determined based on responses from survey respondents since these respondents disposed of the majority of waste generated by them. The waste quantities that had been collected safely, transported safely, and treated safely were estimated. In her previous work, Esseku identified use in animals as one of the categories for generation of waste that requires proper handling and disposal [2]. As this study does not investigate the process of flow in animals, the figures and percentages proposed by Esseku [2] are used in the design of the DDFD for Krowor.

#### 3.3.8. Generation

Generation deals with the various participants in the pharmaceutical value chain who use pharmaceutical products and so have medicines which must be disposed of, where the generation is the generation of pharmaceutical waste. In this study, the figures given by Esseku [2] are adopted as a representative for the different participants: industry contributes eight percent (8%), institutional health care facilities contribute twenty-five percent (25%), pharmacies contribute two percent (2%), community members contribute fifty-five percent (55%), and animal consumption contributes ten percent (10%).

### 3.4. Collection

#### 3.4.1. Pharmaceutical Manufacturers

Collection relates to the proportions of pharmaceutical waste that is safely collected. The relevant data in this regard, includes the ways in which pharmaceuticals are collected from the points of generation. The results of the study show that where pharmaceutical manufacturers have significant quantities of active pharmaceutical ingredients (APIs) or unused and expired pharmaceuticals (UEPs), they hand over all the solid pharmaceutical waste to the FDA for disposal. Effluents from manufacture are treated using WWTPs. With respect to collection in industry, the percentage allocated for safe collection is one hundred percent (100%).

#### 3.4.2. Institutional Care Settings

Institutional care settings in Krowor include hospitals, clinics, and CHPS compounds. The disposal of pharmaceutical waste from these facilities is done with the support of the municipal assembly [9]. The waste is aggregated into hazardous and nonhazardous waste. The nonhazardous waste is collected by waste collectors who are registered with the municipal assembly. Hazardous waste will be collected by specially licenced waste collectors. According to the assembly, these collectors have the requisite knowledge and capacity to handle hazardous waste [9]. This means that the collection of pharmaceutical waste from institutional care settings is done safely.

Liquid waste from institutional care settings is handled by discharge into municipal drain systems. Such waste could contain medicines and active metabolites discharged by attendants and officials in the facilities. Discharge into municipal drainage system will result in receiving waters and surrounding soils becoming exposed to the unchanged medicines or their active metabolites. It must be noted that the institutional health care facilities in Krowor primarily provide ambulatory care [9]. Some of the facilities provide in-patient care and so will have more liquid waste contaminated with unchanged medicines or their active metabolites in the discharge liquid waste. Collection of liquid waste is, thus, not environmentally safe, but only to the extent of contamination by the discharge of attendants, officials, and inpatients, where applicable, into the liquid waste. From the abovementioned, the percentage from institutional care settings that is safely collected is estimated at fifty percent (50%).

#### 3.4.3. Pharmacies

The pharmacies in Krowor comprise mainly of retail outlets with a few doubling as wholesalers of pharmaceutical products. The study shows that of the facilities that have previously disposed of UEPs, seventy-five percent (75%) had handed their solid dosage forms over to the FDA for disposal. Sixty-two percent (62%) of facilities had handed over liquid dosage forms and sixty-nine percent (69%) had done the same with semisolid dosage forms. For these facilities, the collection of these products for disposal is done in an environmentally safe manner.

Twenty-five percent (25%) of respondent facilities indicated that they disposed of their UEPs themselves. Of these, half 12.5% of all respondents indicated that their solid and semisolid dosage forms are added to the general trash and collected for disposal by registered waste collectors. One quarter 6.25% of all respondents of respondent facilities identified this as the method used for the disposal of their liquid dosage forms. These products can, thus, be categorized as being safely collected. The remaining facilities dispose of their UEPs using methods that introduce the products directly into environmental media: open burning and pouring the products down the drain.

From the foregoing, it is estimated that the quantities of pharmaceuticals that are safely collected from pharmacies in Krowor is up to seventy-eight percent (78%).

#### 3.4.4. Households

The study found that at least eighty percent of respondents dispose of their UEPs by adding them to the general trash. This is collected by registered waste collectors [9]. Solid and semisolid dosage forms are more likely to be added to the trash than liquid dosage forms. However, some of the registered waste collectors may not have the means to ensure there is no environmental contamination occurs during the collection process. As Agyepong has suggested, up to sixty percent of individuals and households use waste collectors that may not be able to ensure environmental safety [12]. Respondents indicated that products that are not disposed of in the trash may be poured down the drain, buried with other waste or burnt in the open. From the foregoing, it is estimated that fifty percent (50%) of UEPs with community members in Krowor are safely collected.

#### 3.4.5. Transport

The transport of UEPs from manufacturing sites is under the supervision of the FDA. The aim is to get the UEPs to the disposal sites for the regulated disposal of the product. The transport of these products to the designated disposal sites is generally environmentally safe. With respect to pharmaceutical manufacturers, therefore, it is estimated that one hundred percent (100%) of the products are safely delivered to the disposal sites. Products from institutional care settings, pharmacies, and households are transported by licensed waste collectors. Some of the means used by waste collectors may result in introduction of pharmaceuticals into environmental media. Where head loads and tricycles are used for waste collection [12], some of the waste may end up in the various environmental media as a result of some of the waste blowing off the carriers. With respect to institutional care settings, pharmacies, and households, it is estimated that about fifty percent of the UEPs collected are safely delivered.

### 3.5. Treatment and Reuptake/Reuse

#### 3.5.1. Pharmaceutical Manufacturers

The study showed that pharmaceutical manufacturers hand over APIs and other pharmaceuticals that need disposal to the FDA. The methods used include open burning and crushing and burying for the most part [13]. Where the facilities concerned have incinerators, the FDA supervises the incineration. The study showed that the pharmaceutical manufacturing concerns in Krowor do not have incineration facilities [11]. The FDA may also utilise dilution and discharge into municipal drains when the products requiring disposal are infusions, vitamin preparations, and other such nonhazardous products. These methods may introduce pharmaceuticals into environmental media. The introduction is, however, controlled as the sites are clearly marked out [13].

The study further showed that the respondent manufacturing concerns treat their effluent. Both facilities use WWTPs. These have been found to be unable to remove pharmaceuticals from the wastewater being treated. The methods used for the treatment of pharmaceutical waste from manufacturing concerns in Krowor only provide limited protection for the environmental media. This is because the location of treatment is controlled and diffused as would have if there was indiscriminate discharge. From these assessments, it is estimated that twenty percent (20%) of pharmaceutical waste from manufacturing concerns in Krowor is properly treated.

Results from the study showed that in one of the respondent facilities, the treated water is reused. The analysis mentioned above indicates that the pharmaceutical in the treated effluent is likely to be present in the reused water.

#### 3.5.2. Institutional Care Settings

Solid waste from institutional care settings is disposed of at the engineered landfill in Tema. The overuse of that site will result in improper treatment of the leachate from the waste that is discharged there. As an engineered landfill, there will be treatment of the leachate. This will result in the exposure of receiving waters to the inadequately treated leachate. Liquid waste from these facilities is discharged into municipal drains without any treatment. Any pharmaceuticals that may be present will be discharged directly into the environmental media. Treatment from these settings is estimated as being forty percent (40%) safely done.

#### 3.5.3. Pharmacies

The study shows that some pharmacies utilise open burning mainly for solid and semisolid dosage forms. The primary method of disposal for liquid dosage forms is by pouring the product down the drain. These practices apply to the facilities that dispose of their products without the intervention of the FDA. As earlier discussed, the methods used by the FDA also introduce pharmaceuticals into the environmental media, although to the lower extent. The pharmaceutical waste from these facilities is estimated as by twenty percent (20%) treated safely.

#### 3.5.4. Households

The study shows that ninety-eight percent of individual respondents dispose of their solid and semisolid UEPs with their general waste. On the other hand, eighty-one percent dispose of their liquid UEPs with their general waste. This means on average, about ninety percent of all respondents dispose of their UEPs in the general waste which is collected by the registered waste collectors. This leaves about ten percent of the UEPs disposed of by other means. The means respondents identified as their means of disposal are pouring down the drain, burying in the ground, and open burning. As discussed earlier, these disposal methods introduce pharmaceuticals directly into the environmental media. Disposal by individuals in KroMA is estimated to be zero percent (0%) environmentally safe.

### 3.6. The DDFD for KroMA

**Figure 7:**
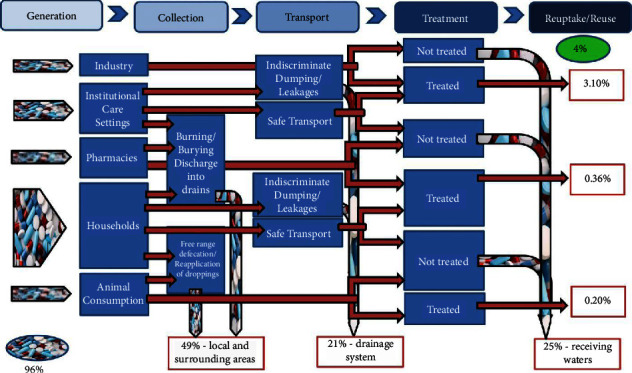
Drug disposal flow diagram showing the likely pathways for disposal of drugs within the Krowor municipality.

The assessed percentages of contributions from various participants in the pharmaceutical value chain were put in a matrix as set out in Table 2. The matrix is then used to produce the DDFD for KroMA as shown in Figure 7. The matrix and the DDFD show that overall, only a little over four percent of the pharmaceuticals are disposed of in a manner that is safe for the environment. Of this, 1.6% is contributed by the industry and 2.5% by institutional care facilities. On the other hand, a little under ninety-six percent of pharmaceuticals end up in the environmental media. Forty-nine percent of disposed pharmaceuticals end up in local and surrounding areas, twenty-one percent in the drainage system, and twenty-five percent in receiving waters. Thus, the soil around the places where the generation of pharmaceutical waste takes place is the most susceptible to contamination by poor disposal practices.

## 4. Discussion

The DDFD for Krowor shows that up to ninety-six percent (96%) of pharmaceutical waste ends up in the environmental media as a result of the handling and disposal practices by stakeholders in Krowor with only four percent (4%) being disposed of safely. It further shows that almost fifty percent (50%) of pharmaceuticals that must be disposed of end up in areas where the waste is generated resulting in contamination of the soils [14]. Targeting the stakeholders who are involved in the generation of the waste and controlling the entry into the environmental media will take care of almost 50% of the contamination. With an estimated 55% of generation being from consumers, strategies such as take back schemes and separation of pharmaceutical waste from the general household waste by consumers will be effective. These strategies will result in improved environmental quality.

Institutions delivering healthcare also contribute up to 27% of the generated pharmaceutical waste. In ambulatory health care facilities, nonhazardous pharmaceutical waste will have to be separated from the municipal waste to control the contamination. It must be noted that all pharmaceutical waste is classified as hazardous by the EPA [15], the practice is to classify some as nonhazardous [9] and dispose of with the municipal waste. Some of the contamination is also contributed by animals defecating free range as well as the reapplication of animal droppings to soil as manure. Improved education of animal farmers will help control the contamination of the environmental media with pharmaceuticals discharged after animals have consumed them.

The DDFD also shows that over twenty percent of pharmaceutical waste ends up in the drainage systems in Krowor. This contamination is mainly due to the means of transport used in the moving of the pharmaceutical waste to disposal sites. The diagram further shows that there is no contamination from pharmaceutical manufacturers. This is because of the controlled way the APIs and UEPs are transported for disposal. This conclusion is based on the assumption that regulations during transportation of waste containing APIs and UEPs are strictly enforced. In addition, this does not account for the final discharge of the transported waste which may contribute to contamination of the environment in other ways. Households, on the other hand, through separation of their pharmaceutical waste and returning of their UEPs in take back schemes, will be able to significantly minimized contamination caused through transportation.

From the DDFD, twenty-five percent (25%) of UEPs end up in receiving waters. This is from the discharge of untreated water into water sources from municipal drains as well as discharge of treated waste with systems that are not designed to remove pharmaceuticals [3, 16, 17]. These may be reduced by education and engagement of stakeholders to understand the impacts, both ascertained and potential, of the impact of poor handling and disposal practices.

## 5. Conclusion

The DDFD provides an effective way of targeting resources to effectively implement EPV strategies for maximum result.

## Figures and Tables

**Table 1:** Disposal methods used by pharmacies located within the Krowor municipality (*n* = 35).

Method of disposal	*Formulation of drug (%)*		
	Solid	Liquid	Semisolid
With general waste	12.5	6.25	12.5
In drain	0	18.75	0
Buried	0	0	0
Open burning	12.5	0	6.25
Handed over to FDA	75	62.5	68.75

**Table 2:** Drug disposal matrix for Krowor municipality.

	Generation (%)	Of which safely collected (%)	Not safely collected (%)	Of which safely transported (%)	Not safely transported (%)	Of which safely treated (%)	Not safely treated (%)	Safe(%)
Industry	8	100	0	100	0	20	80	1.60
		8.00	0.00	8.00	0.00	1.60	6.40	

Institutional care settings	25	50	50	50	50	40	60	2.50
		12.50	12.50	6.25	6.25	2.50	3.75	

Pharmacies	2	78	22	50	50	20	80	0.16
		1.56	0.44	0.78	0.78	0.16	0.62	

Households	55	50	50	50	50	0	100	0.00
		27.50	27.50	13.75	13.75	0.00	13.75	

Animal consumption	10	10	90	100	0	20	80	0.20
		1.00	9.00	1.00	0.00	0.20	0.80	

Unsafe			49		21		25	

## Data Availability

The data used to support the findings of the study are available upon request from the corresponding author.

## Supplementary Materials

Supplementary Materials Respondents ranged in age from sixteen (16) years to seventy-two (72) years of age. The modal and median age range of respondents was twenty-six (26) to thirty (30) years of age which accounted for twenty-three percent (23%) of all respondents participating in the survey. The age bracket of respondents correlates with the age bracket of active persons in the community. Figure SM1 shows the age distribution of respondents. Figure SM1: Age Distribution of respondents. Figure SM2 provides a pictorial presentation of the way in which pharmaceuticals flow into environmental media in Krowor. This provides information on the most cost-effective means of dealing with pharmaceuticals in the environment in the municipality. The process of flow of pharmaceuticals can be described as shown in Figure SM2. This process captures the direction of flow of pharmaceuticals in Krowor. Figure SM2: The process of flow of pharmaceuticals (source: [2]).
