# Supplementary material for: Drug Disposal and Ecopharmacovigilance Practices in the Krowor Municipality, Ghana
Source: J Toxicol. 2022 Dec 30;2022:7674701. doi: 10.1155/2022/7674701 (PMC9822764; doi:10.1155/2022/7674701)
Supplement: Supplementary Materials — Respondents ranged in age from sixteen (16) years to seventy-two (72) years of age. The modal and median age range of respondents was twenty-six (26) to thirty (30) years of age which accounted for twenty-three percent (23%) of all respondents participating in the survey. The age bracket of respondents correlates with the age bracket of active persons in the community. Figure SM1 shows the age distribution of respondents. Figure SM1: Age Distribution of respondents. Figure SM2 provides a pictorial presentation of the way in which pharmaceuticals flow into environmental media in Krowor. This provides information on the most cost-effective means of dealing with pharmaceuticals in the environment in the municipality. The process of flow of pharmaceuticals can be described as shown in Figure SM2. This process captures the direction of flow of pharmaceuticals in Krowor. Figure SM2: The process of flow of pharmaceuticals (source: [2]). [file 7674701.f1.zip › Figure SM2 (1).docx]

Generation

Collection

Transport

Treatment

Reuptake/Reuse

Figure SM2: The process of flow of pharmaceuticals (Source: Esseku, 2015)
